# Bayesian analysis of cytokines and chemokine identifies immune pathways of HBsAg loss during chronic hepatitis B treatment

**DOI:** 10.1038/s41598-021-86836-5

**Published:** 2021-04-02

**Authors:** Sriram Narayanan, Veonice Bijin Au, Atefeh Khakpoor, Cheng Yan, Patricia J. Ahl, Nivashini Kaliaperumal, Bernett Lee, Wen Wei Xiang, Juling Wang, Chris Lee, Amy Tay, Seng Gee Lim, John E. Connolly

**Affiliations:** 1 Translational Immunology Programme, Institute of Molecular and Cell Biology (IMCB), Agency for Science, Technology, and Research, Singapore (A*STAR) Research Entities (RE), 61 Biopolis Drive, Proteos, Singapore, 138673 Singapore; 2 Singapore Immunology Network, A*STAR REs, Singapore, Singapore; 3 IMCB, Tessa Therapeutics Pvt Ltd, Singapore, Singapore; 4 Department of Microbiology and Immunology, Yong Loo Lin School of Medicine, National University of Singapore, Singapore, Singapore; 5 Division of Gastroenterology and Hepatology, Department of Medicine, Yong Loo Lin School of Medicine, National University of Singapore, Singapore, Singapore; 6 Institute of Biomedical Studies, Baylor University, Waco, TX USA

**Keywords:** Diseases, Infectious diseases, Hepatitis, Viral hepatitis, Hepatitis B

## Abstract

Our objective was to examine differences in cytokine/chemokine response in chronic hepatitis B(CHB) patients to understand the immune mechanism of HBsAg loss (functional cure) during antiviral therapy. We used an unbiased machine learning strategy to unravel the immune pathways in CHB nucleo(t)side analogue-treated patients who achieved HBsAg loss with peg-interferon-α(peg-IFN-α) add-on or switch treatment in a randomised clinical trial. Cytokines/chemokines from plasma were compared between those with/without HBsAg loss, at baseline, before and after HBsAg loss. Peg-IFN-α treatment resulted in higher levels of IL-27, IL-12p70, IL-18, IL-13, IL-4, IL-22 and GM-CSF prior to HBsAg loss. Probabilistic network analysis of cytokines, chemokines and soluble factors suggested a dynamic dendritic cell driven NK and T cell immune response associated with HBsAg loss. Bayesian network analysis showed a dominant myeloid-driven type 1 inflammatory response with a MIG and I-TAC central module contributing to HBsAg loss in the add-on arm. In the switch arm, HBsAg loss was associated with a T cell activation module exemplified by high levels of CD40L suggesting T cell activation. Our findings show that more than one immune pathway to HBsAg loss was found with peg-IFN-α therapy; by myeloid-driven Type 1 response in one instance, and T cell activation in the other.

## Introduction

Chronic hepatitis B (CHB) affects over 250 million people globally^1^, and is a major risk factor for development of liver cirrhosis and hepatocellular carcinoma (HCC). HBsAg loss or functional cure of CHB is associated with significant clinical benefits such as reduced HCC and liver complications^2^. The frequency of CHB patients who develop spontaneous HBsAg loss without treatment is very low (1% per year)^3^. A meta-analysis has shown that peg-interferon ± NA is superior to nucleoside analogues (NA) in achieving HBsAg loss^4^.

The immune mechanism(s) related to HBsAg loss are not well established. In acute HBV, resolution of infection is associated with an antiviral T cell response but a poor innate immune response^5^. The prevailing hypothesis is that high antigen load leads to T cell exhaustion in addition to defective adaptive and innate immunity^6^. However, it is unclear how immune clearance of HBsAg occurs, and it is assumed that HBV specific T cells are required to achieve “immune control”. The classic example of immune clearance of HBV occurs in the setting of bone marrow transplantation from a donor with resolved HBV to a CHB patient^7^, where HBV specific T and B cell responses were restored. Studies in acutely infected chimpanzees and transgenic mice models revealed the importance of CD8 T cell responses in HBV control and the finding that HBV can escape innate immune recognition has made the role of innate immunity in CHB ambiguous^8^. In addition, CHB patients have defective B cell response with HBsAg specific B cells showing exhaustion and atypical memory phenotype^9^. Studying the immune mechanisms that lead to functional cure are indeed challenging due to the low frequency of patients achieving functional cure and the assays to evaluate the immune responses are complex and limited by the low frequency of HBV specific T cells^10^. Consequently, there is a significant unmet need to unravel the immune responses associated with functional cure. Since there are limitations to the use of PBMC to assess cellular immunity, and the liver microenvironment requires invasive liver biopsy, one option is to use an array of plasma cytokines and chemokines to interrogate the full spectrum of immune pathways. Cytokines and chemokines exert their effects by modulating the function of immune and non-immune stromal cells. They work in a concerted manner to set off a cascade of immune responses resulting in elimination of viral infection by cell and antibody mediated mechanisms^11^, and the outcome of immune reaction is determined by the quality of cytokine–chemokine response.

To investigate the chemokine-cytokine profile of HBsAg loss, we utilised CHB patients from a randomised clinical trial of NA ± peg-IFN-α (SWAP study) (Suppl. fig. 1A)^12^. We profiled plasma for 87 cytokines, chemokines, growth factors and soluble receptors, pre and post therapy using statistical analysis and Bayesian probabilistic network algorithms to assess the cytokine networks in responders and non-responders.

## Results

### Statistical analysis of chemokines and cytokines

Plasma cytokines were assessed at three time-points during the course of therapy in responders and non-responders. The median time interval from start of peg-IFN-α therapy to loss of HBsAg in responders was 24 weeks (range: 8–72 weeks). The time-point at which the patients were recruited for the study was referred to as the baseline (T0), the time-point before which the responders showed HBsAg loss was labelled T1 (median = 12 weeks) and the time-point after which the responders showed HBsAg loss was labelled T2 (median = 36 weeks) (Supplementary Table 2). Non-responders from similar time-points were chosen for analysis. Comparisons were made within each group over time, and between each group at the matching time-points, using students t-test with corrections for multiple testing and Pearson correlation.

#### Baseline findings in responders and non-responders

**Figure 1:**
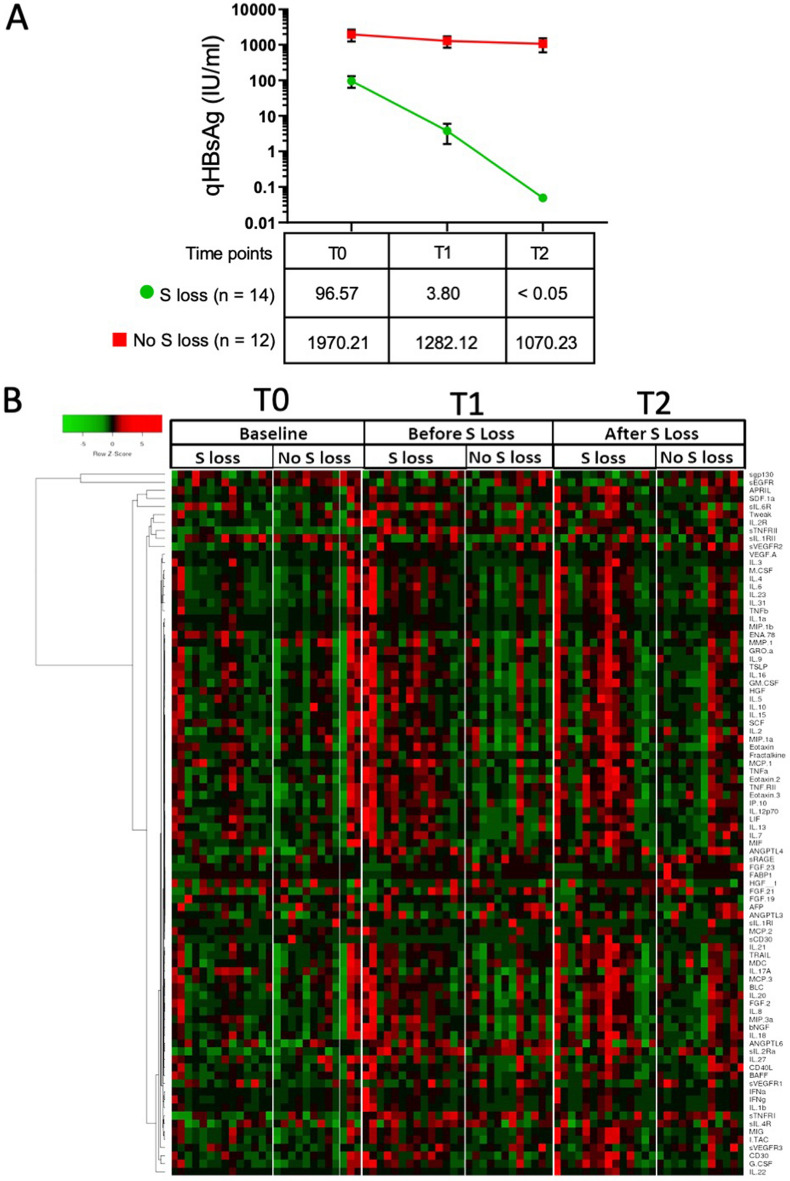
Plasma cytokine levels differ between responders (labelled S loss) and non-responders (labelled No S loss) during peg-IFN-α therapy. (**A**) Trend of quantitative HBsAg (qHBsAg) levels between the responders (S loss) and non-responders (non S loss) in the cohort combined from the Add-on and Switch arms. The numbers below indicate the median value of the qHBsAg for each group at the time-points indicated. (**B**) Heat map of cytokine levels from the plasma samples analysed at the three time-points indicated. The heat map data was clustered on the cytokines and chemokines and separated by time-points. Heat map shows data from responders (S loss; n = 14) and non-responders (No S loss; n = 12). Scale bar indicates the Z score of the normalised cytokine and chemokine values (red, high; green, low).

We observed a difference in qHBsAg at baseline between responders and non-responders where non-responders had 20-fold higher mean qHBsAg levels than responders (Fig. 1A). A clustering analysis and heat map indicated that prior to treatment the cytokine levels were not very different between responders and non-responders (T0, Fig. 1B). Only soluble form of EGFR (sEGFR) was significantly lower at baseline in the responder group. Pearson correlation analyses of plasma levels of HBsAg, with the cytokines and chemokines revealed that the top factors negatively correlated with HBsAg loss were several C–C and C-X-C chemokines together with the cytokines IL-20, IL-12p70, IL-16, IL-18, IL-21, IL-15 and IL-17A (Table 1).

**Table 1:** Pearson correlation analysis of cytokines to HBsAg levels at baseline (T0) time-point.

Cytokine versus HBsAg at baseline	Pearson r	R squared	*P* value (two-tailed)
**Type 1 response**			
IL-12p70	− 0.3985	0.1588	0.0485
IP-10 (CXCL10)	− 0.5055	0.2556	0.0099
I-TAC (CXCL11)	− 0.5046	0.2546	0.0101
BLC (CXCL13)	− 0.5001	0.2501	0.0109
IL-15	− 0.4171	0.174	0.038
IL-18	− 0.4114	0.1692	0.041
MIP-1-alpha (CCL3)	− 0.5576	0.3109	0.0038
MIP-1-beta (CCL4)	− 0.4426	0.1959	0.0267
**T and B cell activation and differentiation**			
CD40L	− 0.4564	0.2083	0.0218
IL-21	− 0.4427	0.196	0.0267
IL-17A	− 0.3974	0.1579	0.0492
APRIL	− 0.4297	0.1846	0.0321
TRAIL	− 0.4689	0.2198	0.0181
IL-16	− 0.4516	0.2039	0.0235
IL-20	− 0.5619	0.3158	0.0035
**Chemokines and other factors**			
MCP-3 (CCL7)	− 0.5239	0.2744	0.0072
Eotaxin-2 (CCL24)	− 0.5172	0.2675	0.0081
Eotaxin (CCL11)	− 0.5089	0.259	0.0094
Eotaxin-3 (CCL26)	− 0.4972	0.2472	0.0114
Fractalkine (CX3CL1)	− 0.4962	0.2462	0.0116
MDC (CCL22)	− 0.421	0.1773	0.0361
MIP-3-alpha (CCL20)	− 0.4783	0.2288	0.0156
TNF-RII	− 0.4123	0.17	0.0405
TSLP	− 0.4549	0.207	0.0223
HGF	− 0.4441	0.1972	0.0262
Tweak	− 0.4432	0.1964	0.0265

Pearson correlation coefficient (r) and R square of cytokines to levels of HBsAg at baseline time-point (T0).

Only cytokines with a two-tailed *P* value less than 0.05 are shown.

#### On-treatment responses

**Figure 2:**
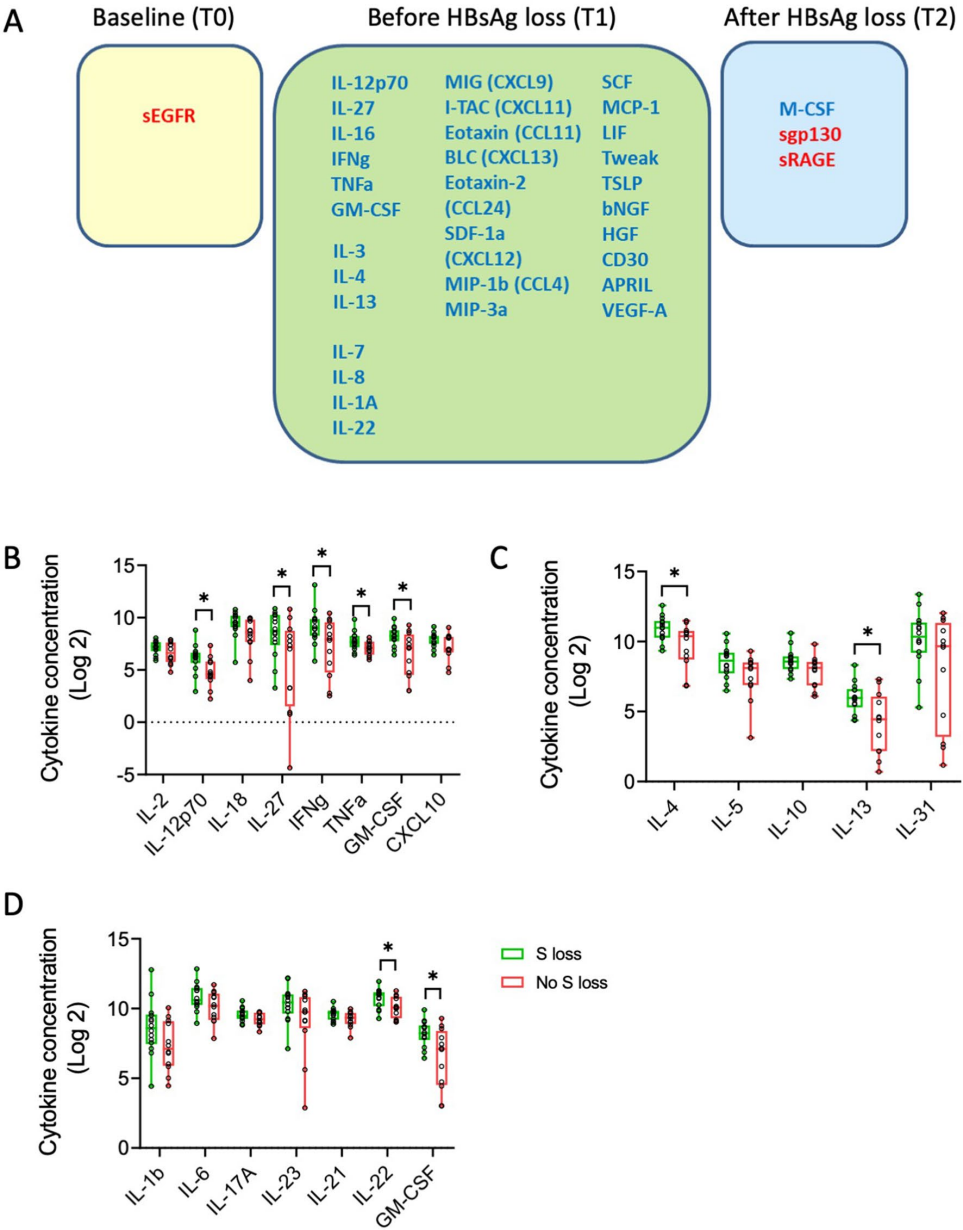
Cytokines that are different between responders and non-responders at the time-points tested. (**A**) Cytokines, chemokines and soluble factors that are significantly increased (blue) or decreased (red) in treatment responders is shown. Statistical analysis was done using t-test. *Indicates *P* value < 0.05 (**B**–**D**) Modular analysis of cytokines and chemokines between responders and non-responders. Bar graphs representing the cytokines and chemokines of (**B**) Type 1 pathway, (**C**) Type 2 pathway, and (**D**) Type 17 pathway at the time-point before loss of HBsAg are shown. *Indicates *P* value < 0.05 from t-test with multiple test corrections.

With peg-IFN-α therapy at the time-point T1 increased levels of several cytokines and chemokines were observed (Fig. 1B; T1) indicating an inflammatory response. This is supported by the elevation in ALT levels before HBsAg loss (Suppl. fig. 1B). The individual cytokines showing an increase at this time-point are shown in Supplementary Figure 2A and B. After HBsAg loss some of these factors were down-regulated (Fig. 1B; T2) indicating resolution of inflammation and further evidenced by decrease in ALT levels in the responders (Supp fig. 1B). These results revealed differences in immune response between responders and non-responders with peg-IFN-α treatment, most notably at the time-point before HBsAg loss (T1). Statistical analysis showed significant increase in cytokine levels in responders compared to non-responders prior to HBsAg loss (Fig. 2A, T1; and Supp fig. 2A and B). When comparing time-points in responders and non-responders there was an increase in sTNFRII, sVEGFR2, sVEGFR3 and sIL-2Ra, while sIL-1RII was found to decrease from T0 to T1 (Suppl Fig. 1C).

#### Peg-IFN-α treatment results in dominant type 1 response in responders

In order to further understand the type of cytokine response, we compared the levels of each analyte between the responders and non-responders. Peg-IFN-α therapy resulted in higher levels of IFN-γ, IL-12p70, IL-7, IL-8, IL-16, IL-4 and IL-13 in the responders (elaborated in next section), before the detectable loss of HBsAg. In addition, chemokines CCL11 (Eotaxin), CCL24 (Eotaxin-2), CCL26 (Eotaxin-3), CCL4 (MIP-1b), CXCL1 (GRO-a), CXCL9 (MIG), CXCL11 (I-TAC), CXCL12 (SDF-1a) and CXCL13 (BLC) were increased in the responders. Taken together, this suggests that a strong inflammatory host response plays a key role in HBsAg loss.

Many of the augmented cytokines/chemokines in responders, which included IL-12p70, IL-27, IFN-γ, TNF-α and CXCL9, are associated with a Type 1 immune response (Fig. 2B). In contrast, among the Type 2 response-associated cytokines only IL-4 and IL-13 were increased in the responders (Fig. 2C). Similarly, among the Type 17 response-associated cytokines, IL-22 and GM-CSF were increased significantly in the responders (Fig. 2D). Taken together, these findings suggest that at the time-point before HBsAg loss, there is a dominance of type 1 immune response in patients who showed HBsAg loss and it probably involves the activation and function of myeloid cells (DCs, macrophages), T cells, NK cells, and B cells.

#### Changes in chemokines and cytokines between timepoints

**Figure 3:**
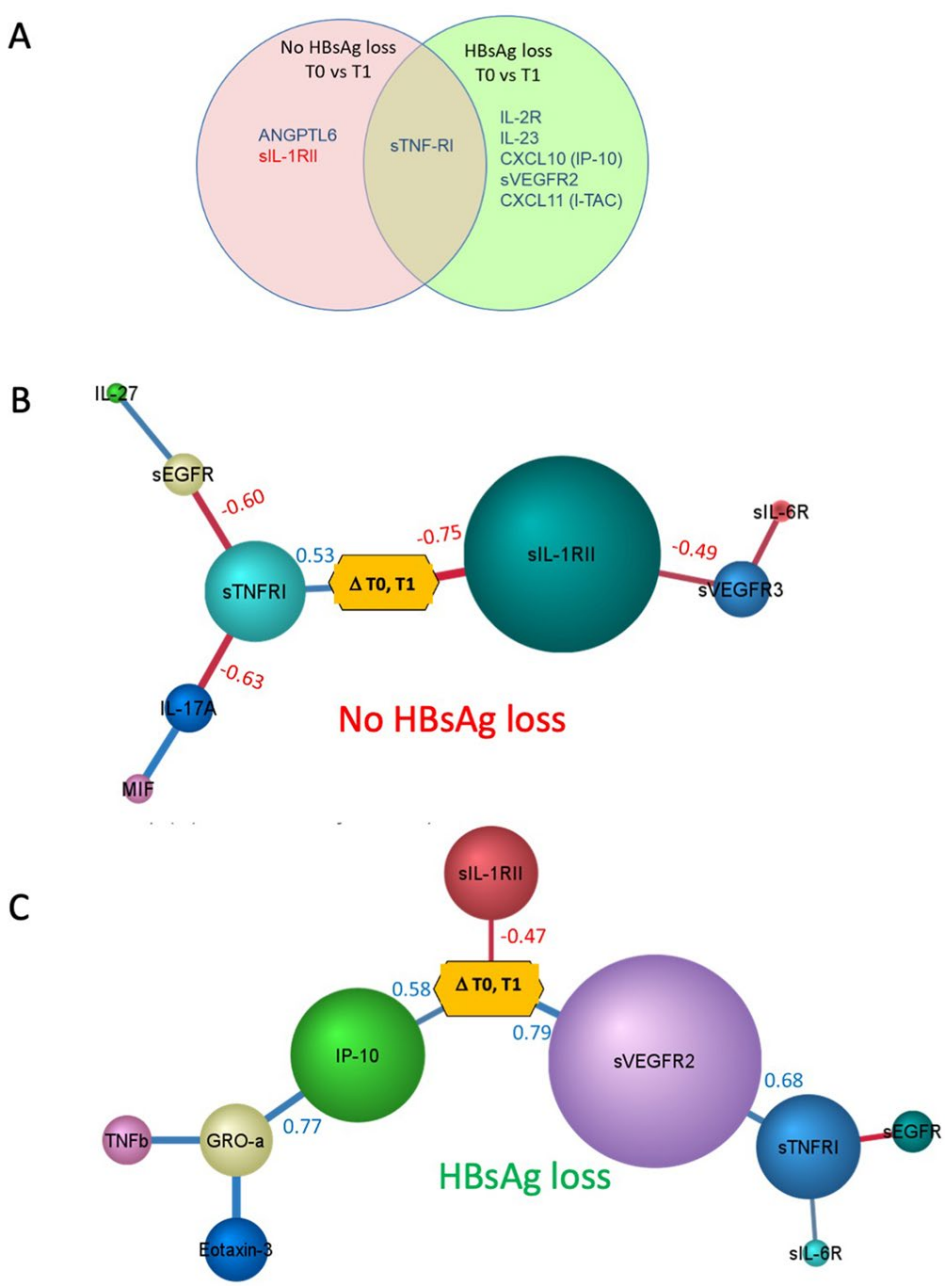
Cytokine differences between time-points in responders and non-responders. (**A**) Venn diagram showing the cytokines that are significantly different between baseline (T0) and before HBsAg loss (T1) time-points. Statistical analysis was done using t-test. Blue and red colour indicates an increase and decrease in levels of cytokine at T1 respectively. (**B**–**C**) Bayesian network of cytokines that are different between the time-points baseline (T0) and before HBsAg loss (T1) in non-responders (**B**) and responders (**C**). Size of the nodes indicate relative contribution to the difference in cytokines or chemokines between the time-points and the colour of the arcs indicate positive (blue) or negative (red) Pearson correlation. The Pearson correlation values for some of the adjoining nodes are shown. Network and figures were generated using BayesiaLab software (version 8) (https://www.bayesia.com/).

To understand peg-IFN-α therapy induced differences in immune response of responders and non-responders, we analysed the differences in cytokines between baseline and before HBsAg loss time-points within each group. A Venn diagram of the cytokines that are significantly different between the two time-points is shown (Fig. 3A). Responders showed increased levels of IL-2R, CXCL10, CXCL11, IL-23 and sVEGFR2. Increase in IL-2R indicates activation of T cells by IL-2. Elevated CXCL10 (IP-10) and CXCL11 suggest a type 1 response. In both groups sTNF-RI increased with IFN-α treatment.

### Bayesian network analysis of cytokines

Cytokines and chemokines act as relay messengers helping immune cells traffic to the site of infection and co-regulate one another. While statistical analyses show the prominent cytokines that are different between responders and non-responders, it fails to account for the complexity of the immune response. However, in a probabilistic network such as Bayesian network, changes in one cytokine/chemokine will affect all other variables in the network. This helps in describing the total effects of cytokines/chemokines in a complex network on the treatment outcome. Therefore, we used Bayesian probabilistic network analysis to examine the contribution of all cytokines in the network with respect to the time-point and with respect to HBsAg loss.

#### Baseline differences between responders and non-responders

**Figure 4:**
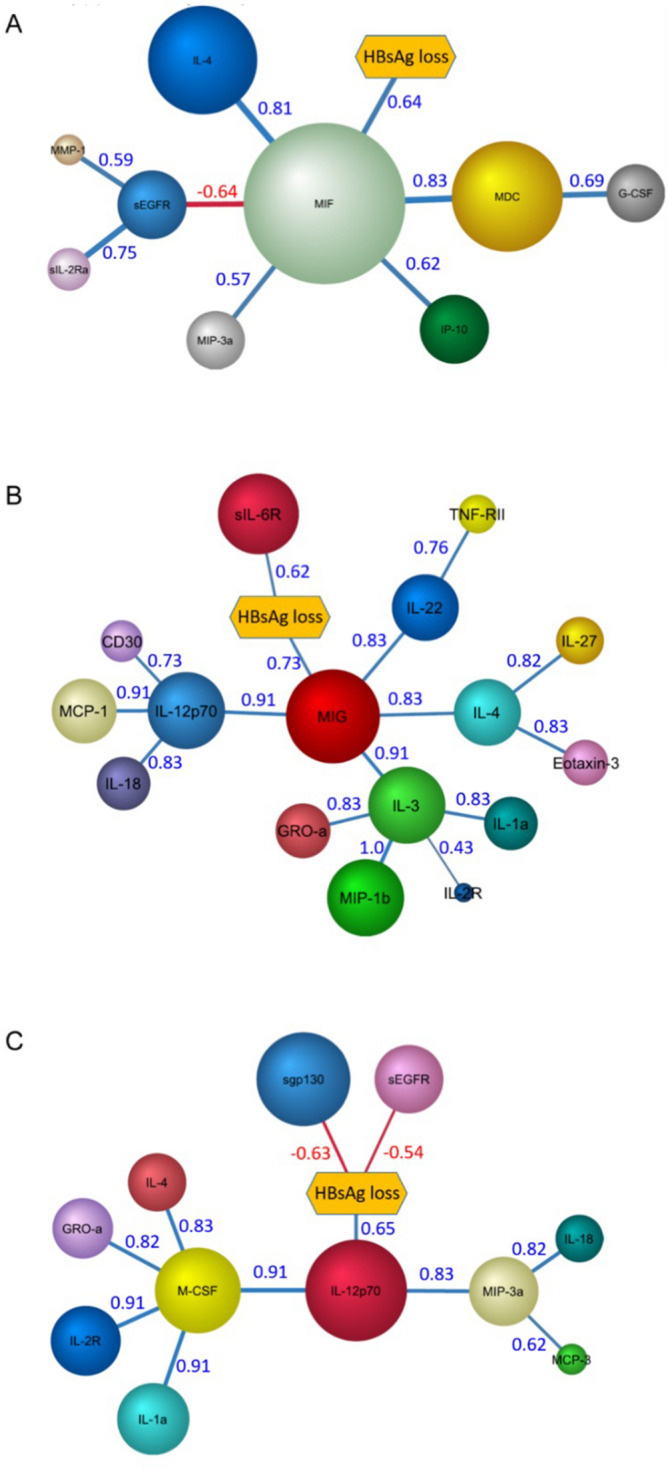
Cytokine network analysis at different time-points during treatment. Bayesian network of cytokines that are associated with responders (patient group) at the baseline time-point (**A**), at the time-point before HBsAg loss (**B**) and after HBsAg loss (**C**). Size of the nodes indicate their relative probabilistic contribution to the loss of HBsAg and the colour of the arcs indicate positive (blue) or negative (red) Pearson correlation between adjoining nodes. The Pearson correlation values for the nodes are as indicated. Network and figures were generated using BayesiaLab software (version 8) (https://www.bayesia.com/).

A semi-supervised tree network of the cytokines at baseline revealed a select number of cytokines were associated with HBsAg loss (Fig. 4A). The size of the node indicates the probabilities of the factors contributing to HBsAg loss. Positive and negative Pearson correlations between the adjoining nodes are indicated by blue and red arcs respectively. Interestingly, a pro-inflammatory module with macrophage migration inhibitory factor (MIF) as the major node was positively correlated with responders in the network at baseline. Other factors like CCL22 (MDC), CXCL10, CCL20 (MIP-3a), IL-4, and G-CSF were positively correlated to MIF. The presence of players from both Type 1 and Type 2 response pathways in the baseline network suggests a lack of clear dominance of any one type of response.

#### Peg-IFN-α treatment induced cytokine differences over time within responders and non-responders

To understand peg-IFN-α therapy induced differences in immune response of the responders and non-responders, we analysed the differences in cytokines between baseline and before loss of HBsAg time-points within each group. Figure 3B and C show the cytokine network differences between time-points T0 and T1 for non-responders and responders, respectively. The levels of sTNFR1 positively correlated with treatment suggesting a TNF-α response in both responders and non-responders. Chemokines CXCL1 and CXCL10 were positively correlated to differences in treatment time-points (ΔT0, T1 in Fig. 3A–B) in the responders suggesting a type 1 immune response. The non-responders did not show any difference for these factors between time-points suggesting that there is dysregulation of response to IFN-α therapy.

#### Enhanced type 1 immune response during peg-IFN-α treatment in responders compared to non-responders

During the course of peg-IFN-α treatment and prior to loss of HBsAg, the network was more complex with many cytokines and chemokines coming into play hinting at an enhanced response in responders (Fig. 4B). The network of cytokines indicated that at the time-point before HBsAg loss, there is an inflammatory response with elevation of many different cytokine modules including Type 1 and Type 2 cytokines. Notably, the HBsAg loss group had increased probabilities of elevated levels of the chemokine CXCL9 and soluble form of the receptor for IL-6 (sIL-6R). The type 1 immunity inducing cytokine IL-12p70 was positively correlated to CXCL9. Furthermore, the cytokines IL-3, IL-22 and IL-4 and chemokines CCL4, CCL2 (MCP-1), CXCL1 and CCL26 showed positive correlations to CXCL9 in the network. These findings indicate the shaping of an enhanced type 1 immune response starting with the activation of DCs, as indicated by increase in IL-12, followed by CXCL9 in recruitment and activation of NK cells and Th1 cells to liver.

The network of cytokines at time-point after loss of HBsAg (Fig. 4C) indicated a dominant type 1 response exemplified by the central node of IL-12p70 that positively correlated to HBsAg loss. The cytokines IL-1a, IL-16, IL-18, M-CSF and IL-4 were positively correlated to loss of HBsAg and associated with the IL-12p70 node in the network. The soluble receptors sEGFR and sgp130 were found to have negative correlation with loss of HBsAg at this time-point similar to that seen at baseline. Chemokines CXCL1, CCL20 (MIP-3a), CCL7 (MCP-3) were also connected to the network. Taken together, these network analyses indicated that with IFN-α treatment the responders showed activation of an inflammatory pathway with the emergence of type 1 response resulting in loss of HBsAg.

### Add-on and switch treatments differ in their cytokine responses

**Figure 5:**
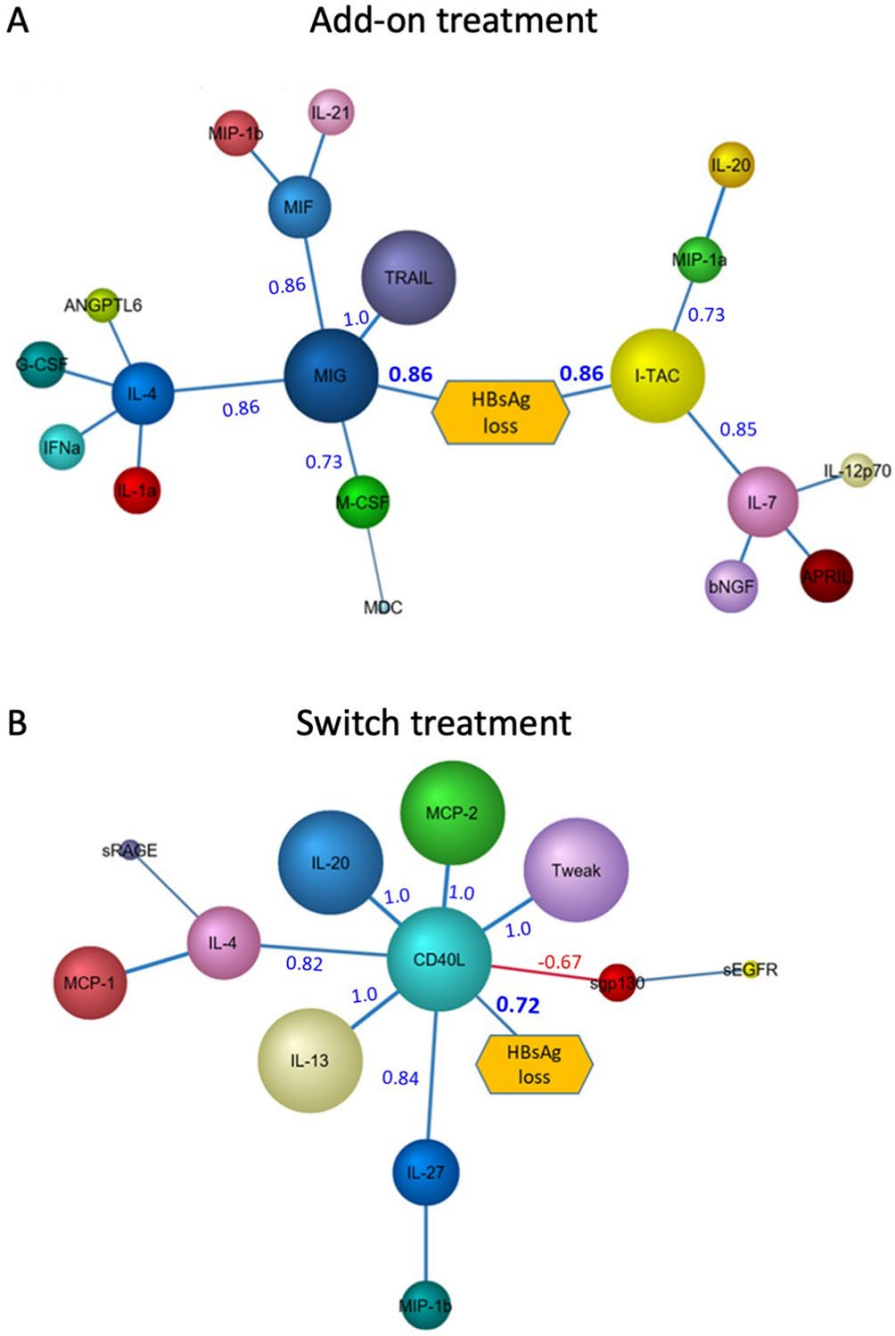
Bayesian network analysis of cytokines at the time-point before loss of HBsAg (T2) in the add-on and switch arms of treatment. Bayesian network of cytokines contributing to loss of HBsAg in (**A**) add-on treatment and (**B**) switch treatment. Size of the nodes indicate relative probabilistic contribution of each node to the loss of HBsAg and the colour of the arcs indicate positive (blue) or negative (red) Pearson correlation between adjoining nodes. The Pearson correlation values for the nodes are as indicated. Network and figures were generated using BayesiaLab software (version 8) (https://www.bayesia.com/).

We next examined the differences in the patient’s immune response between add-on and switch treatment arms. We compared the cytokine levels between responders and non-responders within each treatment arm using Bayesian network analysis. The network of cytokines associating with HBsAg loss in the add-on group is shown (Fig. 5A). Loss of HBsAg positively correlated to CXCL9 and CXCL11. These were the major contributors to HBsAg loss in the network with other cytokines like IL-7, IL-12p70, and IL-4 connected to these two nodes. Other cytokines that contributed to loss of HBsAg via CXCL9 and CXCL11 nodes were TRAIL, IL-7, IL-4, M-CSF, MIF and MIP-1b. The CXCL9 and CXCL11 modules indicated a type 1 immune response with contributions from other cytokines IL-12p70, IL-4, IL-7 and IL-20. The levels of some of the cytokines associated with HBsAg loss from the network are shown (Suppl Fig. 3A). These results indicate a strong DC driven type 1 response typified by the cytokine IL-12p70 and the chemokines CXCL9, CXCL11 in the network.

In the switch treatment arm, however, the cytokine network was less complex compared to that of add-on treatment (Fig. 5B). In this network the only node directly associated with HBsAg loss was CD40L. All other nodes of cytokines like IL-13, IL-4, IL-20, IL-27 and chemokine CCL8 (MCP-2) were connected through the central CD40L node. The levels of some of these cytokines in the responders and non-responders are shown (Suppl Fig. 3B). Soluble TWEAK, a marker of liver inflammation is also present in the network indicating an active inflammation in these patients^13^. These results indicate that the immune response in the switch arm of treatment is probably mediated by activated T cells which may be the result of a Type 2 response. Taken together, these results show that the add-on and switch treatments are quite different in terms of the immune cells and immune responses involved in HBsAg clearance.

## Discussion

The nature of immune responses resulting in HBsAg loss in CHB patients is not well understood. In this study we have employed machine learning and network analysis to unravel the immune responses induced in functionally cured CHB patients treated with peg-IFN-α. Machine learning and network analysis is a powerful method to analyse complex multidimensional data^14, 15^ and has been extensively used for analysing complex data in biology and medicine^16, 17^.

The impact of HBsAg on exhaustion of HBV T and B cell immune responses has been proposed as the reason for the inability to clear HBV^18^, with the duration of exposure to HBsAg and not HBsAg levels, correlating strongly with loss of antigen specific T cell frequency and function^19^. Our findings strongly demonstrate for the first time that low qHBsAg levels at baseline are associated with a pro-inflammatory cytokine and chemokine milieu in the responders, and in particular identified a central role for MIF, a pro-inflammatory marker and an important regulator of innate immunity, is associated with HBsAg loss. MIF was shown to be increased in chronic HBV patients in the immune clearance phase^20^. Bayesian analysis also identified other factors at baseline like CCL22, CXCL11, CXCL10 and IL-4 playing a role in HBsAg loss. These factors associated with MIF in the network play important roles in activation, differentiation and trafficking of effector T cells and NK cells to the site of infection^21–24^. The overall findings indicate that even at baseline the immune milieu was largely pro-inflammatory that set the scene for interferon response.

During peg-IFN-α treatment there was an elevation of inflammatory cytokines and chemokines in responders but not in non-responders suggesting a lack of immune response. This immune response in responders comprised of an increase in several type 1 immune response cytokines (IFN-γ, TNF-α, IL-12p70, and IL-27). IL-12p70 secreted by activated DCs promotes type 1 immune response, and is associated with HBeAg seroconversion during antiviral therapy^25^. Other than a type 1 inflammatory responses, there was evidence of a type 2 inflammatory response with increased IL-4 and IL-13 in the responders before HBsAg loss. IL-4 seems to have an important role as well since it contributes to HBsAg loss in the network at all three time-points. IL-4 has a direct antiviral function by its ability to repress promoters of surface and core antigens of HBV^26^, and is increased in acute self-limiting HBV infection^27^ highlighting its importance in controlling viral infection. The presence of IL-4 in the network indicates a strong multi-functional T cell response. IL-22 enhances infiltration of T, NK and NKT cells to liver and also induces STAT3 activation in liver cells resulting in survival and proliferation of hepatocytes^28–30^. Therefore, IL-22 may play a dual role in enhancing inflammation while mitigating the cellular damage induced by infiltrating immune cells. Consequently, upon peginterferon therapy, the cytokine signature of activation of the myeloid compartment with both type 1 and 2 responses are involved in achieving functional cure. Chemokines play an important role in attracting immune cells into the liver microenvironment to enhance immune responses. In particular, CXCL9, an IFN-γ inducible factor secreted by monocytes and endothelial cells^31^, recruits other lymphocytes to liver during HBV infection^32^ via CXCR3 signalling. This chemokine has been shown to be important in sustained anti-viral response with peg-IFN-α treatment^33^.

Separate Bayesian analysis of add-on vs switch arm of treatment revealed a differential cytokine/chemokine network involved in HBsAg loss. In patients who had add-on peginterferon therapy, the dominant feature was MIF, TRAIL and the CXCL9-CXCL11 module. These findings support a dominant type 1 response. In the switch arm however, soluble CD40L was the dominant node indicating T cell activation mediated by IL-4, IL-13, IL-27 and IL-20. CD40L is a co-stimulatory receptor expressed on activated CD4 T cells and plays an important role in activation of B cells and other APCs by binding to CD40^34^. Our data suggests that the functional cure is achieved by more than one immune mechanism with the involvement of both type 1 and type 2 responses, and is probably mediated by non-cytolytic function of cytokines^35–37^. A dominant myeloid module was observed to be active in the add-on arm, while the switch arm showed T cell activation.

Immune responses are seldom due to one particular cell type, cytokine or chemokine but occur as a network response. We have utilized the power of Bayesian analysis in identifying these immune response modules rather than individual cytokines or chemokines. We have further shown that Type 1 and Type 2 networks are both involved in orchestrating an immune response leading to HBsAg loss. Consequently, we can conclude that there are multiple immune networks and more than one immune mechanism involved in HBsAg loss. Further interrogation of these networks using PBMCs and liver samples is needed to determine the immune mechanism of HBsAg loss. Finally, we show that Bayesian probabilistic analysis is an unbiased machine learning approach that provides insights into immune responses leading to HBsAg loss, the hallmark of functional cure in CHB.

## Methods

### Patients and samples

In this randomised control study^12^, CHB patients were enrolled to three different arms of therapy, as shown in Fig. 1A and Supplementary Fig. 1A. Out of the 202 patients receiving NA switched to peg-IFN-α, or NA+ peg-IFN-α, 17 patients achieved HBsAg loss (8%). Patients in the switch arm stopped NA 4 weeks after randomisation while in the add-on arm, NA was continued till the end of study at week 72. The study was approved by the National Healthcare Group Domain Specific Review Board, Singapore (NHG DSRB Ref: 2016/00713). The research was performed in accordance with all relevant regulations, and informed consent was obtained from all participants and performed in accordance with the Declaration of Helsinki. The study was registered in clinicaltrials.gov (NCT01928511) on 26/08/2013. A total of 26 patients (12 patients without HBsAg loss (non-responders) and 14 with HBsAg loss (responders) were then selected. Plasma samples collected at three time-points (baseline, before HBsAg loss and after HBsAg loss) were analysed. HBsAg loss was defined as a negative result on the ARCHITECT HBsAg Qualitative II assay (Abbott Diagnostics, Abbott Park, IL, USA) at week 72 of the study.

### Multiplex cytokine and chemokine analysis

Plasma samples were analysed for a panel of cytokines, chemokines and soluble factors (Table 2). A total of 87 analytes (Supplementary Table 1) were measured using 3 multiplex panels; Immune Monitoring 65-plex ProcartaPlex Panel (Thermofisher, Waltham, Massachusetts, USA), Human Soluble Cytokine Receptor Panel and Human Liver Protein Panel (Merck Millipore, Billerica, MA, USA) (Supplementary Table 1). Assays were performed as per manufacturer's instructions. Plates were washed using Tecan Hydrospeed Washer (Tecan, Männedorf, Switzerland) and read with Flexmap 3D system (Luminex Corp, Austin, TX, USA). Data were analyzed using the Bio-Plex manager 6.2 software with a 5-parameter curve-fitting algorithm applied for standard curve calculations.

**Table 2:** Grouping of cytokines and chemokines based on the type of immune response.

Cytokine group	Cytokines/chemokines
Pro-inflammatory	IL-1-beta, IL-1-alpha, IL-2, IL-6, IL-12, IL-18, IL-27, IFN-gamma, TNF-alpha, GM-CSF
Anti-inflammatory	IL-4, IL-13, IL-10, IL-11, TGF-beta
Type 1	IL-2, IL-12, IL-18, IL-27, IFN-gamma, TNF-alpha, CXCL10 (IP-10), CXCL9 (MIG)
Type 2	IL-4, IL-13, IL-5, IL-31, IL-33, IL-27
Type 17	IL-6, IL-17A, IL-21, IL-22, GM-CSF
B cell factors	APRIL, BAFF, IL-2, IL-4, IL-6, IL-10

### Data normalization and statistical analysis

Cytokine concentrations were log transformed and normalised for plate variations using *ComBat* from Bioconductor package sva based on a plasma reference sample that was run along with samples in all the plates^38^. Data normalisation was confirmed using PCA analysis of data from different batches/plates. Only normalised values were used for analysis and reported in this manuscript. Differences in plasma cytokine concentrations between groups were calculated using the student t-test and corrected for multiple comparisons using Bonferroni’s method where indicated in the figure legends. Associations of cytokine concentrations with clinical parameters were determined using Spearman Rank correlations. Graphpad prism (version 8) and R software version 3.1.2 were used for statistical computation^39^. Heatmaps were generated using Heatmapper (www.heatmapper.ca)^40^.

### Bayesian probabilistic network analysis

Probabilistic machine-learning network analysis was performed using BayesiaLab software (version 8) (https://www.bayesia.com/). Data discretization was performed using supervised multivariate approach with the parameter HBsAg loss as target node. Data were discretized into two bins and analysed using optimised structural co-efficient (alpha) values for each time-point. Networks were generated by semi-supervised approach using Maximum Weight Spanning Tree algorithm along with Taboo order post processing to ensure robust network generation. The networks were then graphed for the probability contribution of nodes to the target node and the Pearson correlation values between adjoining nodes. For the networks looking at responders and non-responders, pooled data from both treatment arms were used. The HBsAg loss status was assigned as the target node and the network was constructed around it as described above. For analyses of responders and non-responders in the two treatment arms, networks were generated using data from each treatment arm separately. Similar workflow was used for generating networks using time-points as target nodes for analysis of treatment-induced changes between responders and non-responders (Delta T0, T1).

## Supplementary Information

Supplementary Information.

## Abbreviations

CHB: Chronic hepatitis B
HCC: Hepatocellular carcinoma
NA: Nucleos(t)ide analogue
IFN-α: Interferon-α
peg-IFN-α: Pegylated interferon-α
ALT: Alanine transaminase
DC: Dendritic cells
