# Supplementary material for: Bayesian analysis of cytokines and chemokine identifies immune pathways of HBsAg loss during chronic hepatitis B treatment
Source: Sci Rep. 2021 Apr 2;11:7455. doi: 10.1038/s41598-021-86836-5 (PMC8018960; doi:10.1038/s41598-021-86836-5)
Supplement: Supplementary file 1 — Supplementary Information. [file 41598_2021_86836_MOESM1_ESM.docx]

**Bayesian analysis of chemokine/cytokines identify immune pathways of HBsAg loss during Chronic Hepatitis B treatment**

Sriram Narayanan^1^, Veonice Bijin Au^1^, Atefeh Khakpoor^5^, Cheng Yan^5^, Patricia J. Ahl^1^, Nivashini Kaliaperumal^1^, Bernett Lee^2^, Wen Wei Xiang^3^, Juling Wang^5^, Chris Lee^5^, Amy Tay^5^, Seng Gee Lim*^1,5^, John E. Connolly*^1,4,6^

Supplementary Figures and Tables

1. Supplementary Figure 1
2. Supplementary Figure 2
3. Supplementary Figure 3
4. Supplementary Table 1
5. Supplementary Table 2

**Supplementary Fig. 1**

**
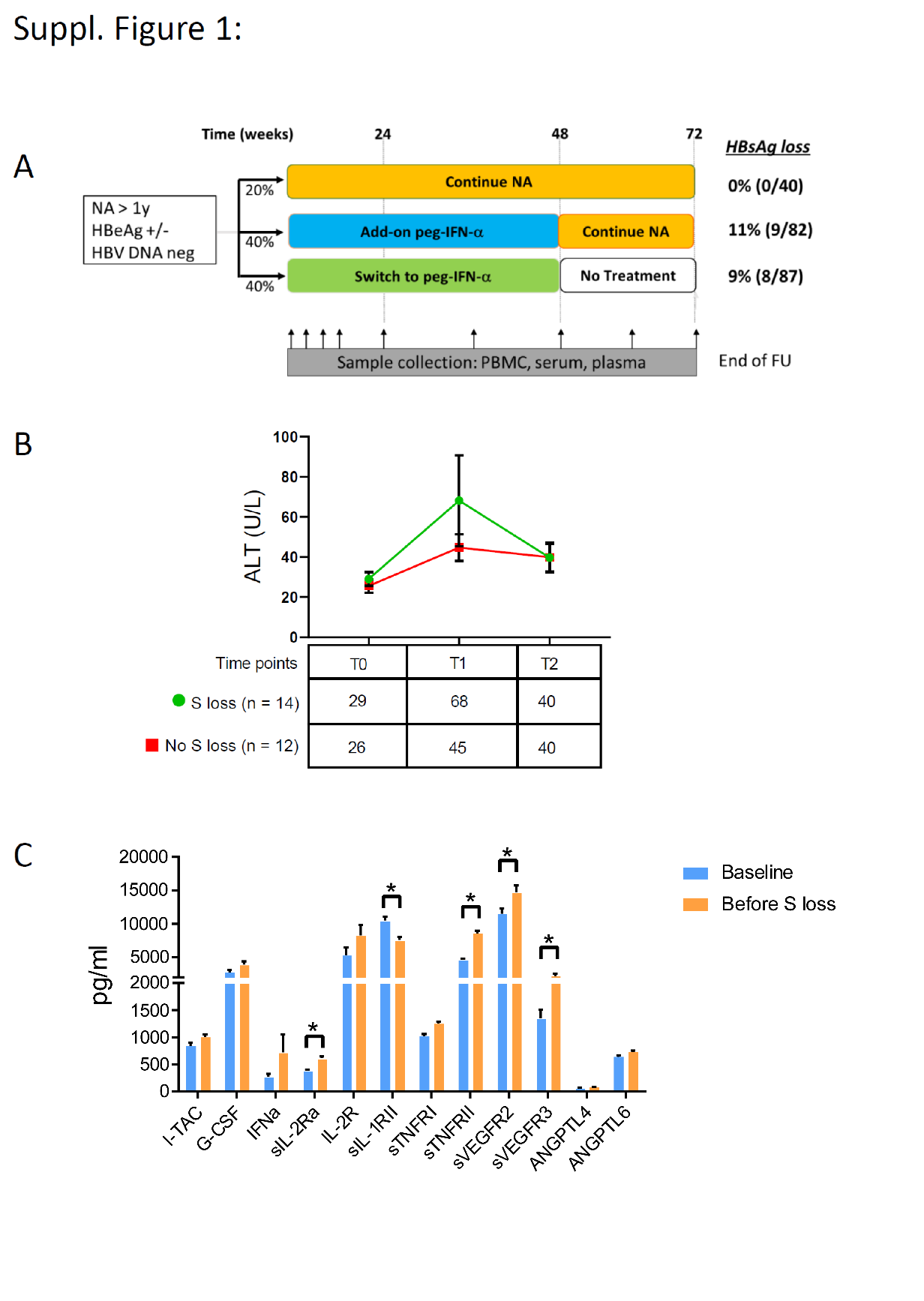
**

**Supplementary figure 1:** (**A**) Treatment arms of SWAP cohort and the sample collection time-points are shown. NA refers to nucleos(t)ide analogue treatment and peg-IFN-α refers to pegylated interferon-α 2b. The time is indicated in weeks on the top and the sample collection points are indicated by arrows at the bottom. (**B**) Trend of ALT levels in the responders (S loss) and non-responders (non S loss) in the cohort combined from the Add-on and Switch arms at the time-points indicated. The numbers below indicate the median value of ALT (U/L) for each group at the time-points indicated. (**C**) Comparison of differences in chemokines or soluble factors between the baseline and before HBsAg loss time-points. Statistical analysis was done using student’s t-test with multiple test corrections. * indicates p values < 0.05.

**Supplementary Fig. 2A**

**
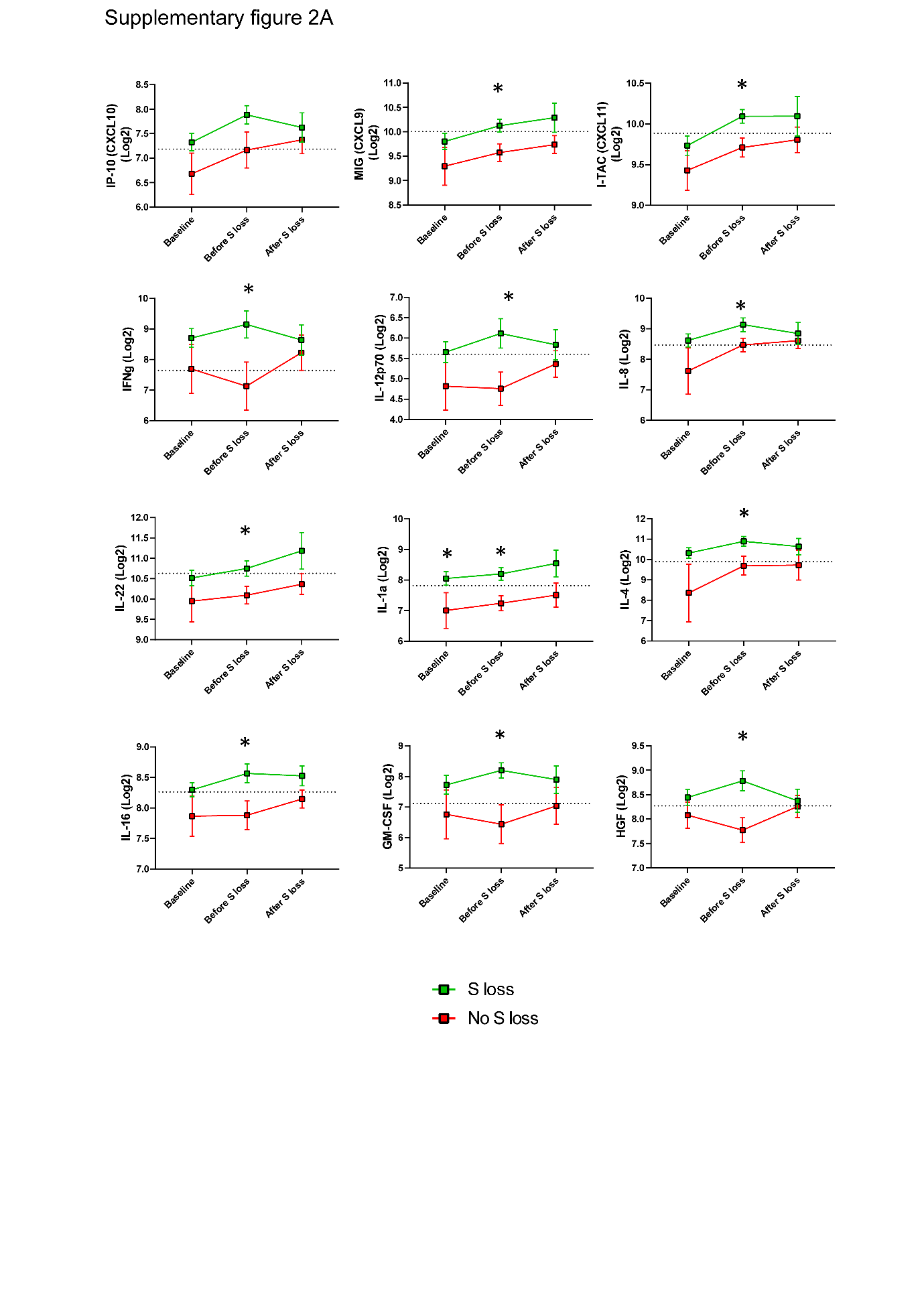
**

**Supplementary figure 2A:** Kinetics of cytokines that show significant difference at the before HBsAg loss time-point (T2). Dotted line indicates mean cytokine levels from healthy donors. * indicates statistical significance by t-test (p < 0.05) for the corresponding time-point between patients showing HBsAg loss and patients showing no loss of HBsAg.

**Supplementary Fig. 2A continued**

**
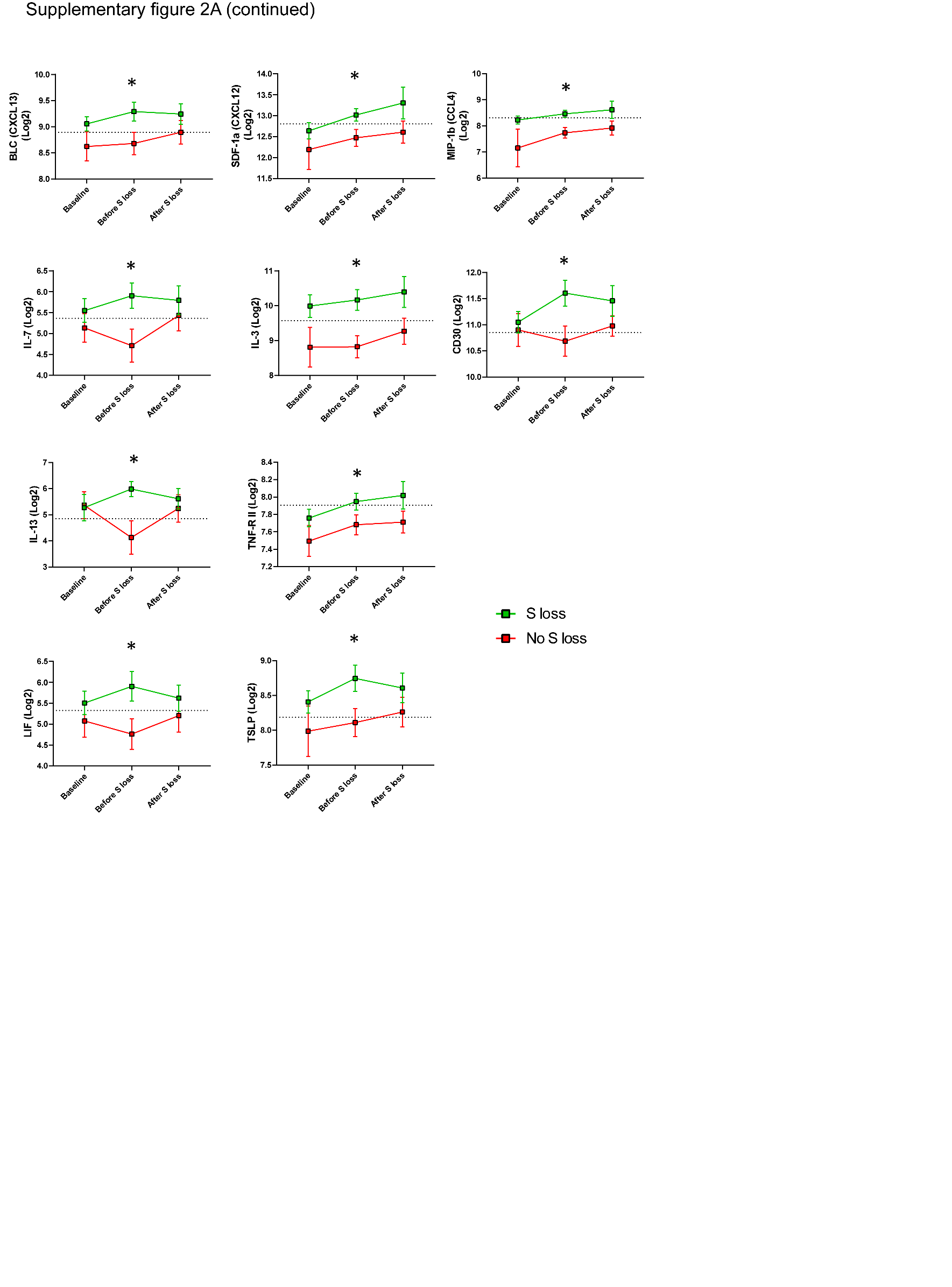
**

**Supplementary Fig. 2B**

**
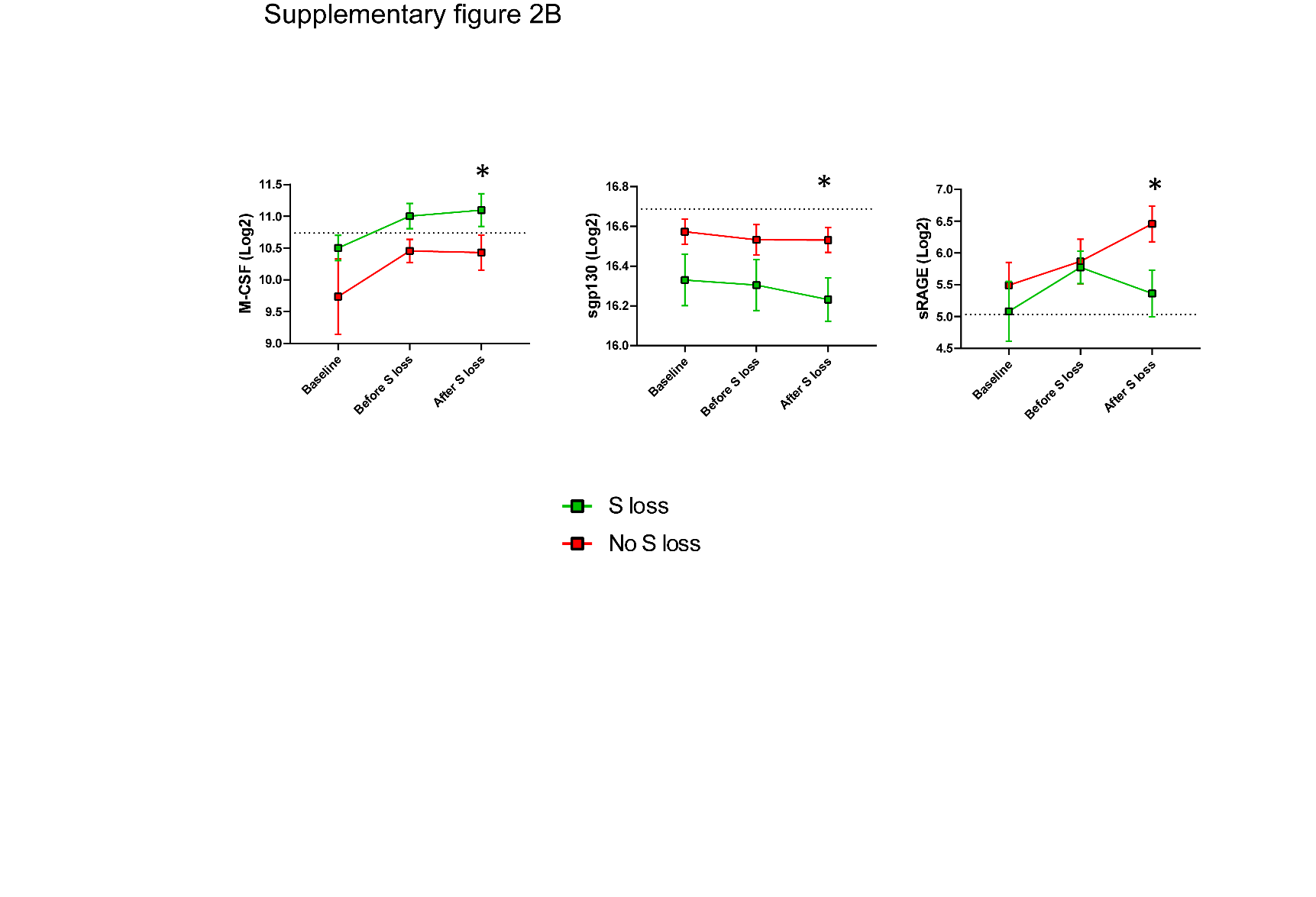
**

**Supplementary Fig. 2B:** Kinetics of cytokines that show significant difference at the after HBsAg loss time-point (T3). Dotted line indicates mean cytokine levels from healthy donors. * indicates statistical significance by t-test (p < 0.05) for the corresponding time-point between patients showing HBsAg loss and patients showing no loss of HBsAg.

**Supplementary Fig. 3**

**
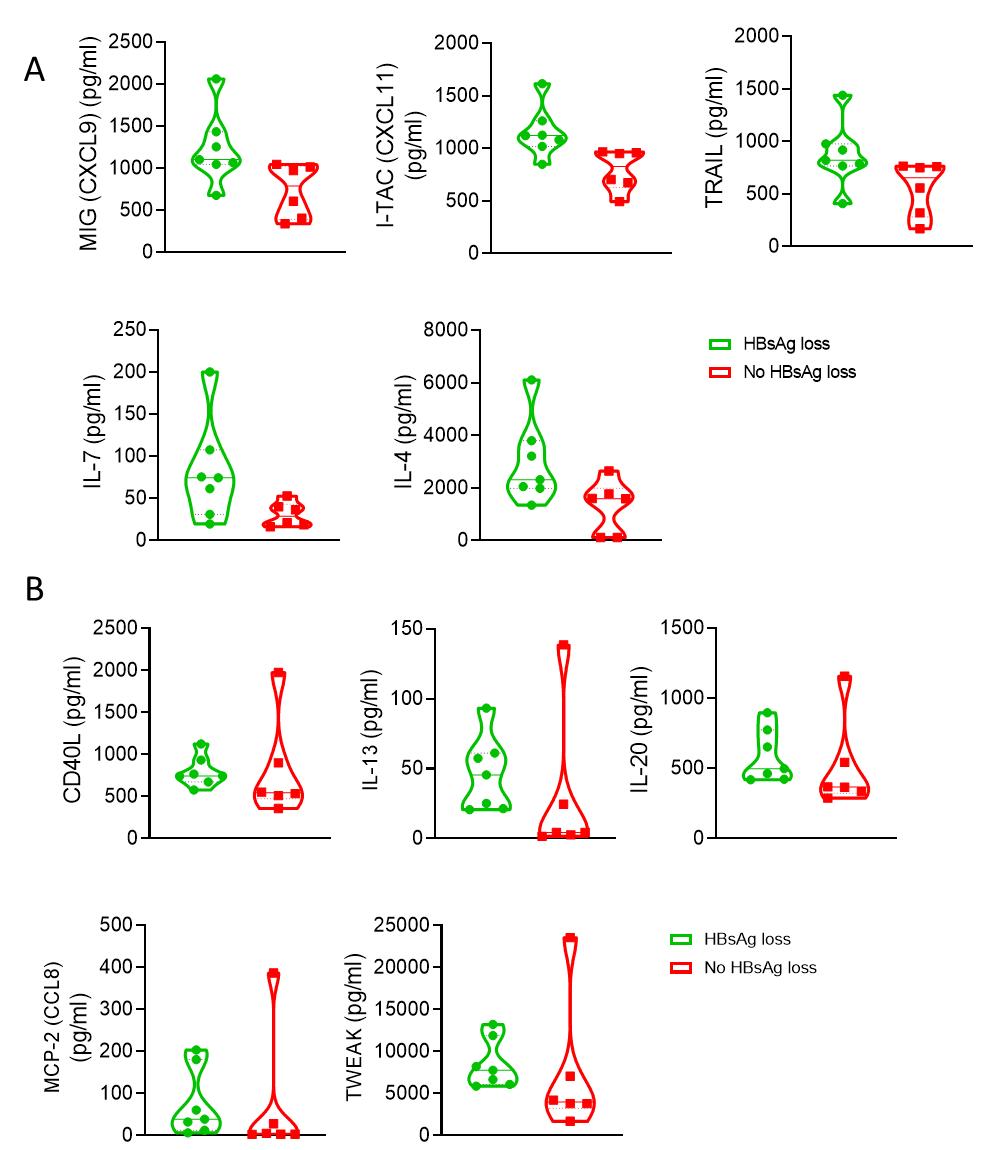
**

**Supplementary figure 3*:*** *Levels of cytokines from the bayesian network with differences in responders and non-responders (refers to Figure 5).* (**A**) Levels of cytokines and chemokines in the responders and non-responders from add-on arm bayesian network and (**B**) from switch arm bayesian network.

**Supplementary Table 1**. List of analytes quantified in this study.

| **Interleukins** | **Cytokines** | **Chemokines** | **Growth factors** | **Soluble receptors** | **Liver markers** |
| --- | --- | --- | --- | --- | --- |
| IL-10 | G-CSF | BLC (CXCL13) | ANGPTL3 | APRIL | AFP |
| IL-12p70 | GM-CSF | ENA-78 (CXCL5) | ANGPTL4 | BAFF | FABP1 |
| IL-13 | IFNa | Eotaxin (CCL11) | ANGPTL6 | CD30 | HGF |
| IL-15 | IFNg | Eotaxin-2 (CCL24) | bNGF | CD40L |  |
| IL-16 | LIF | Eotaxin-3 (CCL26) | FGF-19 | IL-2R |  |
| IL-17A | M-CSF | Fractalkine (CX3CL1) | FGF-2 | MMP-1 |  |
| IL-18 | MIF | GRO-a (CXCL1) | FGF-21 | TNF-RII |  |
| IL-1a | SCF | IP-10 (CXCL10) | FGF-23 | TRAIL |  |
| IL-1b | TNFa | I-TAC (CXCL11) | VEGF-A | Tweak |  |
| IL-2 | TNFb | MCP-1 (CCL2) |  | sCD30 |  |
| IL-20 | TSLP | MCP-2 (CCL2) |  | sEGFR |  |
| IL-21 |  | MCP-3 (CCL7) |  | sIL-1RI |  |
| IL-22 |  | MDC (CCL22) |  | sIL-1RII |  |
| IL-23 |  | MIG (CXCL9) |  | sIL-2Ra |  |
| IL-27 |  | MIP-1a (CCL3) |  | sIL-4R |  |
| IL-3 |  | MIP-1b (CCL4) |  | sIL-6R |  |
| IL-31 |  | MIP-3α (CCL20) |  | sRAGE |  |
| IL-4 |  | SDF-1 (CXCL12) |  | sTNFRI |  |
| IL-5 |  |  |  | sTNFRII |  |
| IL-6 |  |  |  | sVEGFR1 |  |
| IL-7 |  |  |  | sVEGFR2 |  |
| IL-8 |  |  |  | sVEGFR3 |  |
| IL-9 |  |  |  | sgp130 |  |

**Supplementary Table 2:**

| **No. of responders** | **Wk 4** | **Wk 8** | **Wk 12** | **Wk 24** | **Wk 36** | **Wk 48** | **FU Wk 12 (Wk 60)** | **FU Wk 24 (Wk 72)** | **FU Wk 48 (Wk 96)** |
| --- | --- | --- | --- | --- | --- | --- | --- | --- | --- |
| HBs Ag loss  time-point | 0 | 1 | 2 | 6 | 1 | 3 | 0 | 1 | 0 |
| Before HBsAg loss (T1) | 4 | 0 | 5 | 1 | 4 | 0 | 0 | 0 | 0 |
| After HBsAg loss (T2) | 0 | 0 | 0 | 3 | 6 | 1 | 3 | 0 | 1 |

**Supplementary Table 2:** Time-points of HBsAg loss in the responders. Table showing the number of responders (n = 14) showing HBsAg loss at different time-points during the treatment and the follow-up period (FU). The time-points are in weeks from the start of peg-IFNα therapy. The table also indicates the number of responders at time-points chosen as before HBsAg loss (T1) and after HBsAg loss (T2) time-points.
